# Supplementary figures and images for: Reg-1α, a New Substrate of Calpain-2 Depending on Its Glycosylation Status
Source: Int J Mol Sci. 2022 Aug 2;23(15):8591. doi: 10.3390/ijms23158591 (PMC9369050; doi:10.3390/ijms23158591)

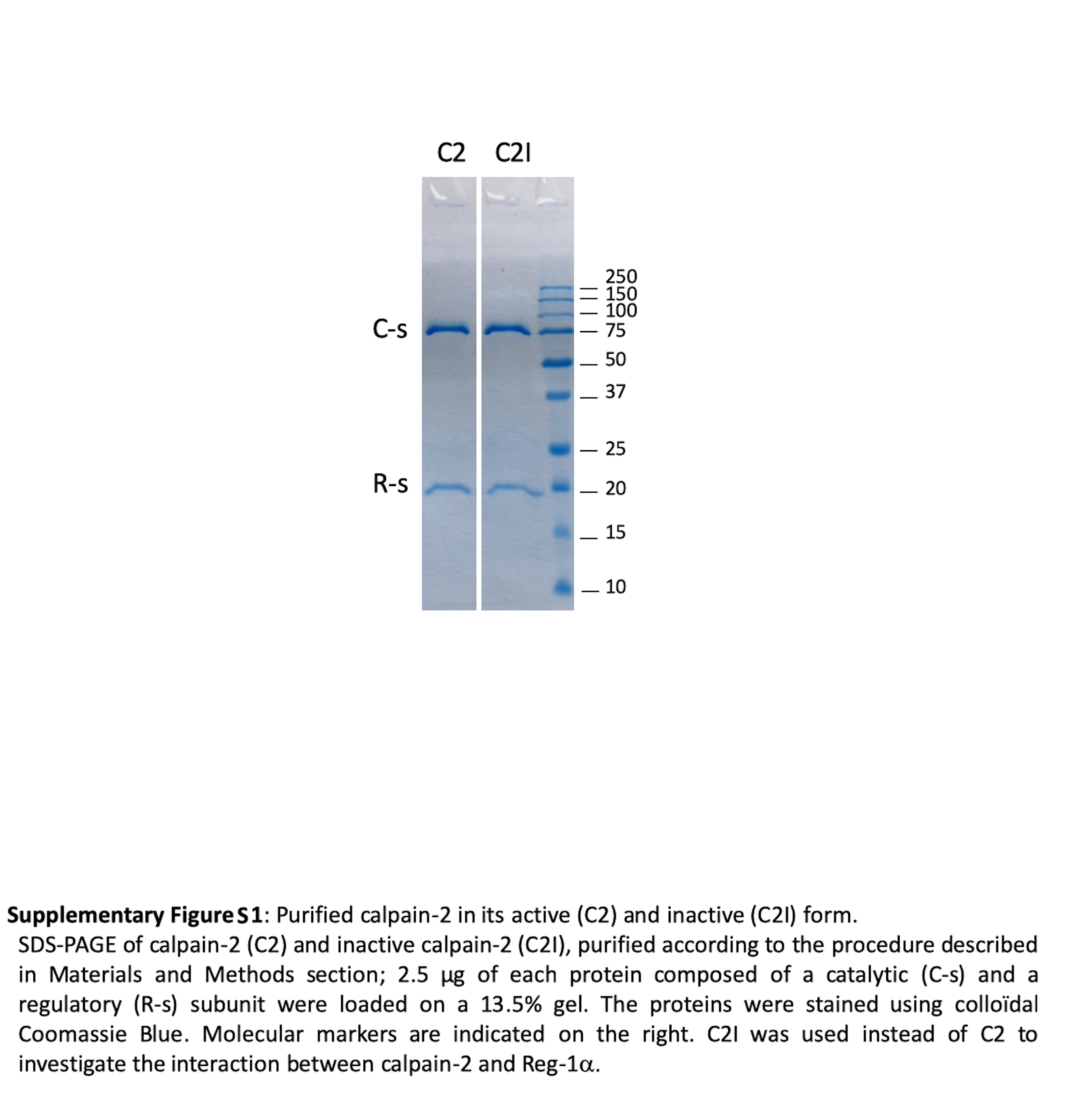

Supplement: Supplementary file 1 [file ijms-23-08591-s001.zip › Figure S1_Lebart.tiff]

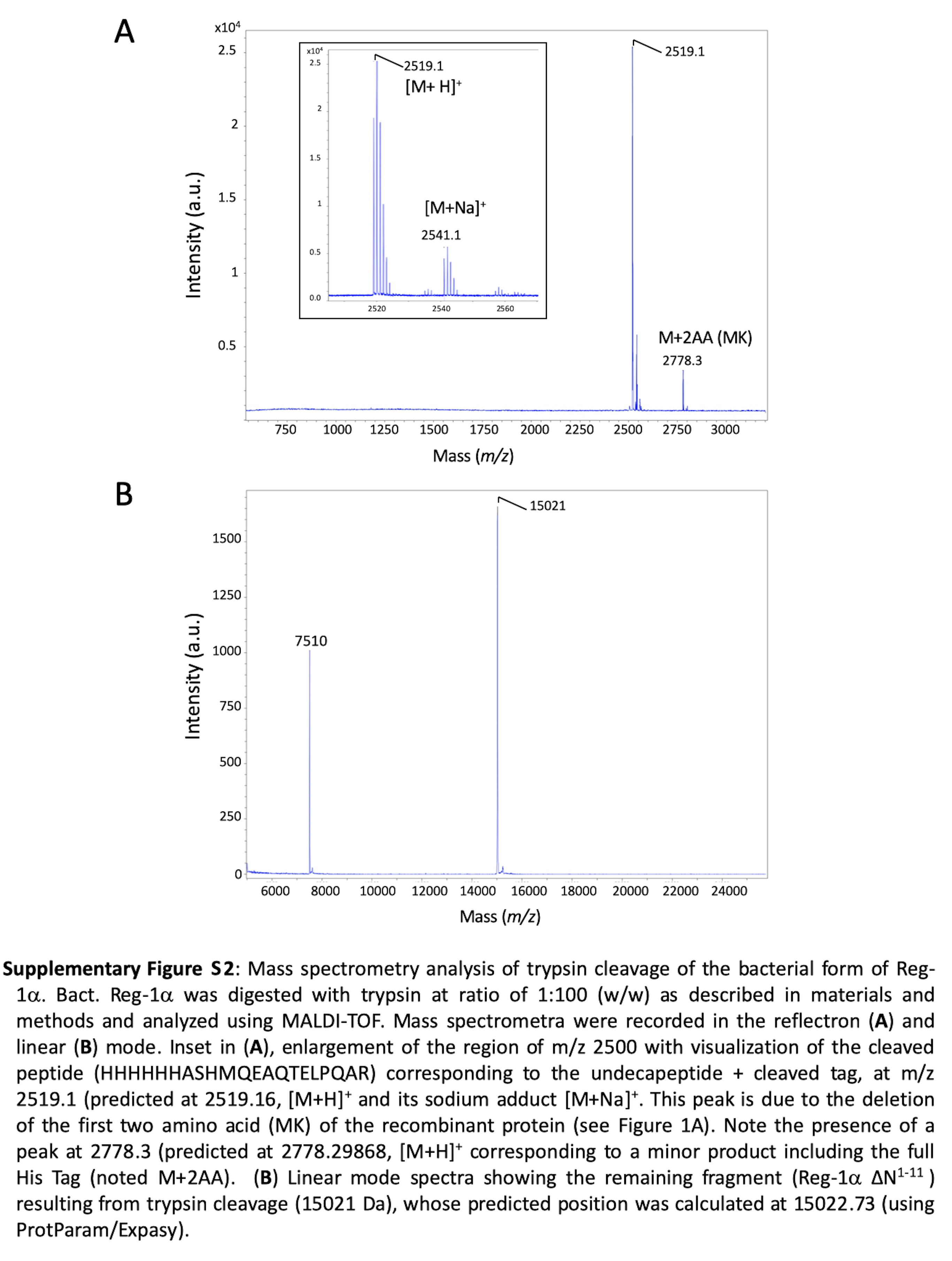

Supplement: Supplementary file 1 [file ijms-23-08591-s001.zip › Figure S2_Lebart.tiff]

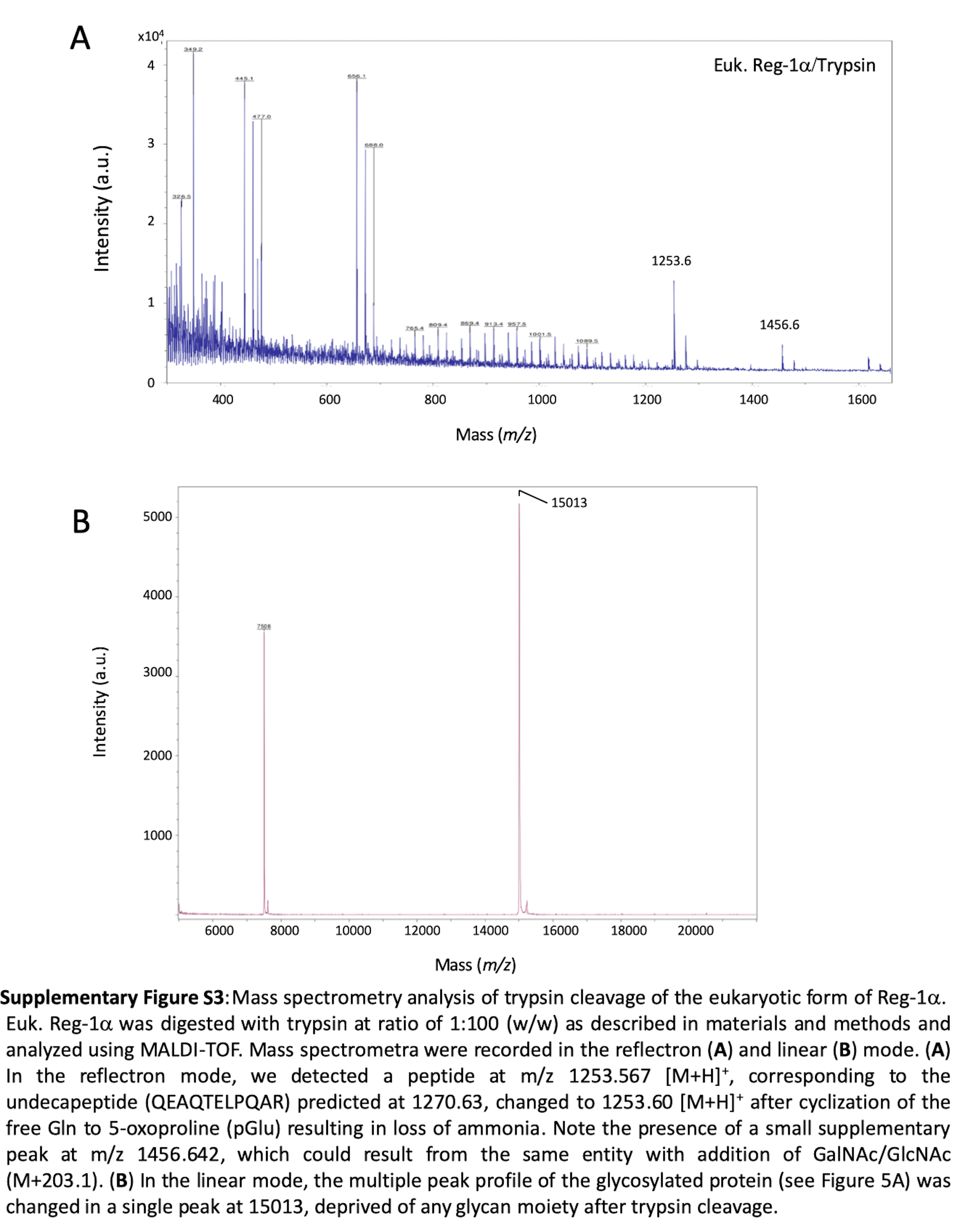

Supplement: Supplementary file 1 [file ijms-23-08591-s001.zip › Figure S3_Lebart.tiff]

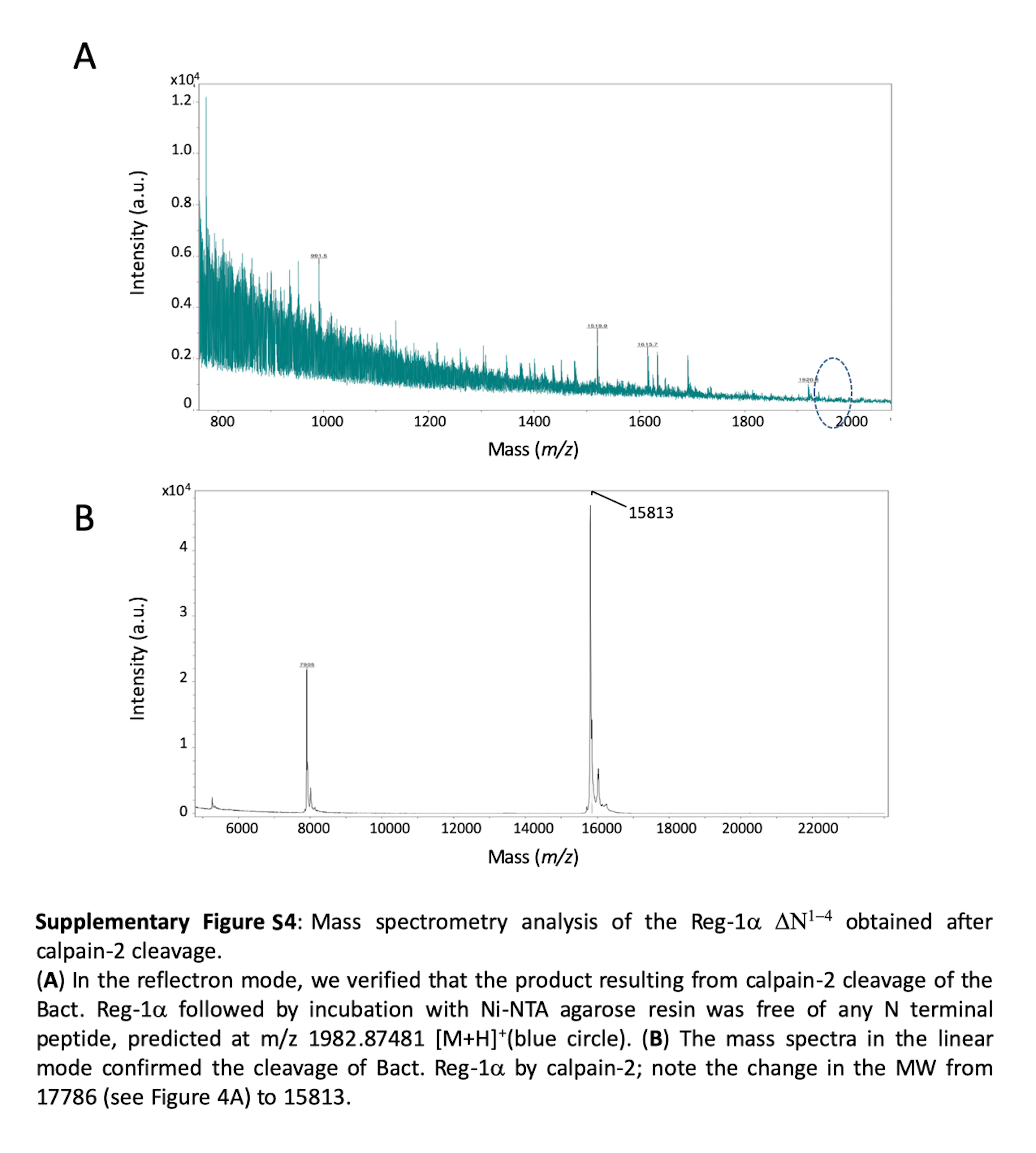

Supplement: Supplementary file 1 [file ijms-23-08591-s001.zip › Figure S4_Lebart.tiff]

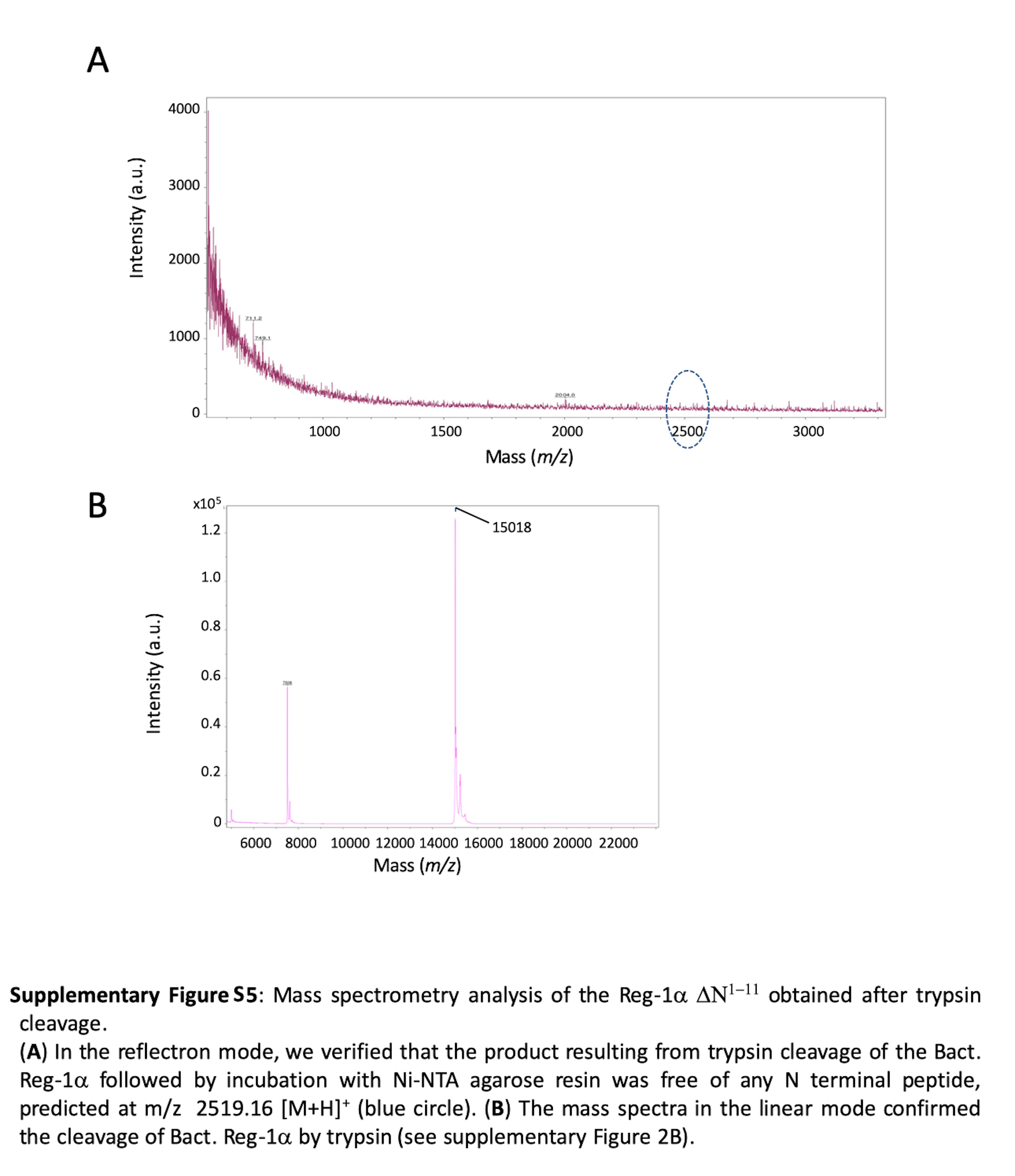

Supplement: Supplementary file 1 [file ijms-23-08591-s001.zip › Figure S5_Lebart.tiff]
